# Classification of lung adenocarcinoma transcriptome subtypes from pathological images using deep convolutional networks

**DOI:** 10.1007/s11548-018-1835-2

**Published:** 2018-08-29

**Authors:** Victor Andrew A. Antonio, Naoaki Ono, Akira Saito, Tetsuo Sato, Md. Altaf-Ul-Amin, Shigehiko Kanaya

**Affiliations:** 1 Graduate School of Science and Technology, Nara Institute of Science and Technology, Ikoma, Japan; 2 Data Science Center, Nara Institute of Science and Technology, Ikoma, Japan; 3 Division for Health Service Promotion, University of Tokyo, Tokyo, Japan; 4 Graduate School of information Science, Nara Institute of Science and Technology, Ikoma, Japan

**Keywords:** Deep learning, Lung cancer, Computer-aided diagnosis, Autoencoder, Independent subspace analysis

## Abstract

**Purpose:**

Convolutional neural networks have become rapidly popular for image recognition and image analysis because of its powerful potential. In this paper, we developed a method for classifying subtypes of lung adenocarcinoma from pathological images using neural network whose that can evaluate phenotypic features from wider area to consider cellular distributions.

**Methods:**

In order to recognize the types of tumors, we need not only to detail features of cells, but also to incorporate statistical distribution of the different types of cells. Variants of autoencoders as building blocks of pre-trained convolutional layers of neural networks are implemented. A sparse deep autoencoder which minimizes local information entropy on the encoding layer is then proposed and applied to images of size $2048\times 2048$. We applied this model for feature extraction from pathological images of lung adenocarcinoma, which is comprised of three transcriptome subtypes previously defined by the Cancer Genome Atlas network. Since the tumor tissue is composed of heterogeneous cell populations, recognition of tumor transcriptome subtypes requires more information than local pattern of cells. The parameters extracted using this approach will then be used in multiple reduction stages to perform classification on larger images.

**Results:**

We were able to demonstrate that these networks successfully recognize morphological features of lung adenocarcinoma. We also performed classification and reconstruction experiments to compare the outputs of the variants. The results showed that the larger input image that covers a certain area of the tissue is required to recognize transcriptome subtypes. The sparse autoencoder network with $2048 \times 2048$ input provides a 98.9% classification accuracy.

**Conclusion:**

This study shows the potential of autoencoders as a feature extraction paradigm and paves the way for a whole slide image analysis tool to predict molecular subtypes of tumors from pathological features.

## Introduction

Recent rapid development of machine learning algorithms brings us a wide range of applications for image recognition and classification. In particular, a significant advancement of visual recognition using deep learning architectures has been shown by the ImageNet Large-Scale Visual Recognition Challenge (ILSVRC) [16], which has served as a testbed for a few generations of large-scale image classification systems. A convolutional neural network (CNN) provides a promising architecture that can extract features from given images automatically, optimize the manifold of image space, and show great success in image classification and medical image analysis [17, 20]. In this study, we propose an application of CNNs for feature extraction and classification of lung adenocarcinoma pathological images and use the learned features for classification of large image data.

These approaches use small images as input, usually less than 300 px by 300 px. However, whole slide images gathered by the Cancer Genome Atlas (TCGA) network are of a much larger magnitude. This paper presents an approach that transfers information learned from small input images to larger input data. By applying unsupervised learning through autoencoders, we will be able to extract features that are not heavily reliant on classification information [5, 7].

Specifically, this project has two goals. First, it aims to compare the performance of different network architectures in reconstruction and visualization tasks. To do this, we conduct experiments using several types of networks on lung cancer images gathered from the Cancer Genome Atlas database (http://cancergenome.nih.gov) as an example training database. Second, we developed the extended network to process much larger input of pathological images, in order to evaluate not only local phenotypic features but also their distribution in the tissue, in order to apply the deep convolutional autoencoders, for the classification of lung adenocarcinoma images into their transcriptome subtypes, with the understanding that modifying existing machine learning methods to target specific image sets can optimize the precision and accuracy of the analysis.

## Review of previous work

### Classification of lung adenocarcinoma transcriptome subtypes

**Fig. 1:**
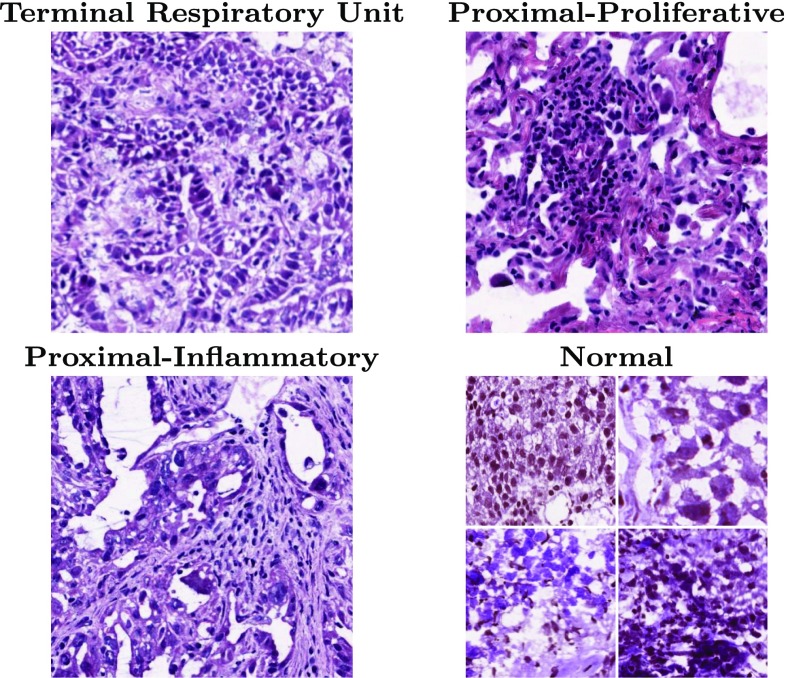
Pathological images of three lung adenocarcinoma subtypes

Lung cancer is the leading cause of cancer-related mortality, and adenocarcinoma is its most common histological subtype [8, 9]. The overall prognosis for lung cancer remains poor, despite recent advances in molecular targeted therapies. Several cancer genome projects have analyzed cohorts of lung cancer patients and revealed genome and transcriptome alterations. Most recently, the Cancer Genome Atlas (TCGA) has described the comprehensive genomic landscape of lung adenocarcinoma in a large cohort [18]. These studies not only elucidated oncogenic mechanisms but also shed light on previously unappreciated heterogeneity of gene expression profiles. As a consequence of genomic alterations and gene mutations in cancer cells, aberrant patterns of gene expression profiles occur, which eventually determine cancer cell behaviors. The pathological images from resection are paired with transcriptome data from DNA microarray for each patient. In line with this, it is worth noting that the aforementioned TCGA study has identified three transcriptome subtypes of lung adenocarcinoma: the terminal respiratory unit (TRU, formerly bronchioid), the proximal-proliferative (PP, formerly magnoid), and the proximal-inflammatory (PI, formerly squamoid) transcriptional subtypes [19]. It has been further demonstrated that this classification is associated with clinical features and gene mutation profiles. In terms of morphological features, lung adenocarcinomas display high inter-individual and intra-tumoral heterogeneity. However, it remains undetermined whether the transcriptome subtypes are associated with distinctive patterns of pathological findings. If it is the case, image analyses on biopsy of resected tissue samples will be helpful to infer transcriptional changes in tumor tissues, which can assist precise diagnosis and clinical decision making. In this study, we propose a model to classify three lung adenocarcinoma transcriptome subtypes from their pathological images using a deep learning approach. Figure 1 shows a sample of each of the three subtypes alongside four different samples of normal images.

### Image processing via CNNs

Machine learning continues to be a vital innovation used in several fields. From a biology standpoint, it can be used for gene expression interpolation and classification of several datasets. Moreover, it has been an important instrument for image classification and inference in recent years. On the other hand, image classification and analysis has been an important achievement of computational systems in recent times, and in fact, it is still a growing, revolutionary field. Specifically, being able to perform analysis on pathological images proves to be vital for medicine and bioinformatics. Image processing methods using deep neural networks are currently developing very rapidly. However, those approaches mainly target general image analysis such as photo-classification and face recognition. On the other hand, an analysis of biomedical images requires a more specific viewpoint focusing extensively on their biological features [2, 12, 14, 15].

This study relies on an investigation conducted by Masci et al. [13] to determine the ability of standard CNNs to extract features using an unsupervised learning method. They used the MNIST dataset, which has been the standard for this type of testing. We see in this work the ability of pre-training a network for reconstruction to still be capable of performing classification. They were able to extract features from unlabeled data, which when combined with backpropagation algorithms can still become efficient classifiers.

We also constructed a deep learning model of the sparse autoencoder (SAE) for the differentiation of the distinct types of lung adenocarcinoma from pathological images [3, 21].

Finally, we see from [11] a specific type of CNN using the notion of a reconstruction independent subspace analysis (RISA). This is an unsupervised learning method for the reconstruction of images emphasizing the invariance between the extracted features, which means that neighboring filters are designed to share the same property. They were able to show that these invariant features are vital in the classification of images if we attach a supervised layer to the pre-trained RISA network.

Since part of our goal is an understanding of the internal architectures of several CNN variations, we also look at [22]. Their work provides a number of visualization experiments for this purpose, and we can follow a similar approach for our data.

Finally, machine learning and computer vision has provided us powerful methods to improve accuracy and efficiency of image classification. These methods rely upon manually curated image features to characterize specific features of tumors. However, recent development of approaches like deep neural networks allows us to extract image features from given data automatically, without using handmade features. Using pre-trained neural networks, we can extract features of tumors and distinguish them according to their shapes. However, when we address the classification of adenocarcinoma subtypes, local features of cell shapes are not enough to describe the variation and distribution of various cells in the tissue [10]. In this paper, we propose variations of CNNs that uses multiple reduction layers in order to evaluate a large area of pathological images and classify lung adenocarcinoma subtypes.

## Model

### Autoencoders

**Fig. 2:**
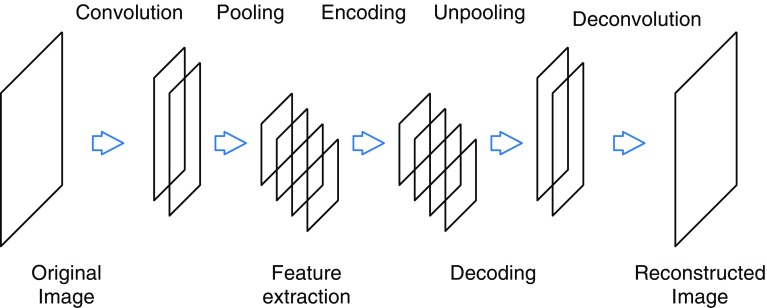
Autoencoder model based on a convolutional neural network

An autoencoder is an unsupervised machine learning architecture that extracts characteristic features from given inputs by learning a network which reproduces input data from those features. Figure 2 shows the basic design of the autoencoder used in our model. The input data are scanned by a convolutional filter and down-sampled by a max-pooling layer then passed on to an encoding layer. The output here can then be used to generate the input data using the reversed network. The total network is optimized to minimize the difference between input and output data.

**Fig. 3:**
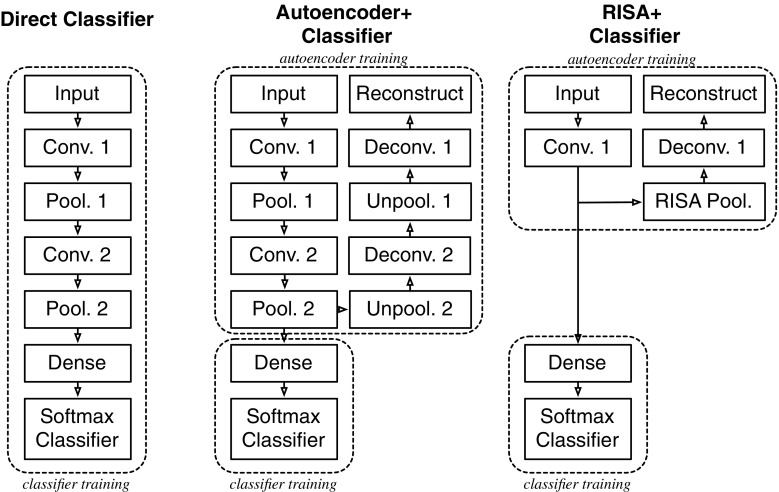
Pipelines for classifier variants. Conv.: convolution layer with $5 \times 5$ filters. Pool.: pooling layers with $2 \times 2$ max pooling. Dense: fully connected layers. Unpool: unpooling by copying to $2 \times 2$ pixels. Deconv.: deconvolution with the same size of filters

To enhance the efficiency of feature extraction and information compression in the autoencoder, we introduced a sparsity penalty. We compute information entropy of the output of the encoding layer and add the penalty for the optimization function (*L*) to minimize the effect of overfitting. The optimization function is defined as follows:

1 $$\begin{aligned} L=R+\lambda _{s}S, \end{aligned}$$

where

2 $$\begin{aligned} R=\sum _{i}^{N}\left( x_{i}^{\text {output}}-x_{i}^{\text {input}}\right) ^2 \end{aligned}$$

and

3 $$\begin{aligned} S=\sum {k=1}^n\sum _{j=1}^{M}\left( -r_{j}^{\text {encode}}\log r_{j}^{\text {encode}}\right) . \end{aligned}$$

Here, $r_{j}^{\text {encode}}$ is the output intensity of filter *j* in the encoding layer relative to their total summation. *N* and *M* are the numbers of nodes in the input and encoding layers, respectively, and $\lambda _{s}$ is a weight constant.

Stacked autoencoders allow us to extract more complex image features with higher-order structures, while some detail information will be lost in down-sampling. It is worth noting that stacked autoencoders can be trained independently. That is, the network of the first autoencoder can be fixed after training and left aside when we train the network for the second optimizer. This reduces the number of trainable parameters and required computation.

### Classifier variants

In the first part of this study, we implemented three types of classifiers and compared their corresponding results. These networks can be distinguished based on how the convolutional filters will be learned and extracted.

We call the first network a direct classifier, and it is described by a convolution network attached to a softmax classification layer. The features will be extracted according to optimal classification. Softmax cross-entropy will be used as a loss function. The subsequent networks are pre-trained autoencoder CNNs. The final layer of these networks will be attached to a softmax classification layer, and its features will be extracted similar to the direct classifier.

Particularly, the second network is a pre-trained autoencoder whose features are extracted following the reconstruction paradigm *R* from Eq. (2).

On the other hand, the third network is a pre-trained reconstruction independent subspace analysis (RISA) network. It is a two-layer autoencoder variant composed of convolution and pooling layers. The main distinction of a RISA network is that it emphasizes minimal translational invariance [11]. If we denote the learned matrix from the convolutional layer as *C*, and the fixed matrix from the pooling layer as *H*, then for an input vector $\mathbf {x}$, the second layer output is

$$\begin{aligned} p_{i}\left( \mathbf {x};C,H\right) =\sqrt{\sum _{m=1}^{k}H_{im}\left( \sum _{j=1}^{n}C_{mj}\mathbf {x}_{j}\right) ^{2}}. \end{aligned}$$

The features extracted from a RISA network will be learned through the following heuristic:

$$\begin{aligned} \begin{aligned} \arg \min _{C}\sum _{t=1}^{N}\left( \frac{1}{N}\left\| CC^{\mathrm {T}}{\mathbf {x}}^{\left( t\right) }-{\mathbf {x}}^{\left( t\right) }\right\| ^{2}+\lambda \sum _{i=1}^{k}p_{i}\left( {\mathbf {x}}^{\left( t\right) };C,H\right) \right) , \end{aligned} \end{aligned}$$

where $\left\{ \mathbf {x}^{\left( t\right) }\right\} _{t=1}^{N}$ is the input dataset and $\lambda$ a weight constant.

This rule extracts features less expensively than manually designed feature extraction methods.

Figure 3 shows a summary for the different pipelines for the three variants. Here, the softmax classifier takes logistic outputs.

### Toward classification of larger images

**Fig. 4:**
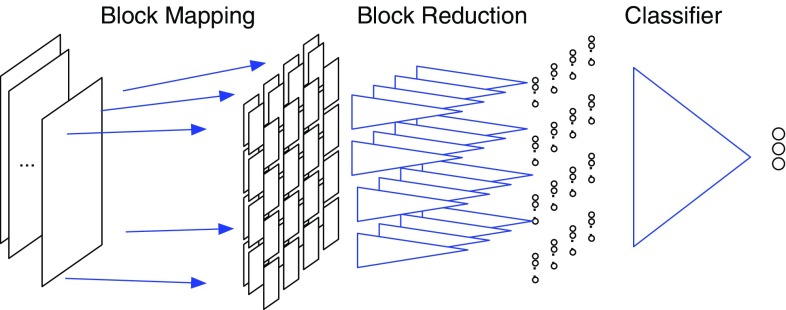
Structure of the whole network

In the second part of this study, we constructed a model based on three autoencoders and one classification reducer that takes logistic outputs. Figure 4 shows the structure of the network. $2048\,\hbox {px} \times 2048\,\hbox {px}$ slices from the pathological images were used as input for the first autoencoder. For an initial feature extraction, we first pre-train three stages of convolutional autoencoder. The output from the encoding layer of the third autoencoder is passed to the reduction classifier. Since the size of the third encoding layer is still large, we divided it into $16 \times 16$ subpanes, and in each subpane, the input from the encoding layer is reduced to 24 output nodes through fully connected networks. Note that all the subpanes share the same reduction network; in other words, it is also a convolution without overlap between windows. Finally, the output of the reduction layer is reduced again into three nodes which represent the three classes of lung adenocarcinoma subtypes. Using multiple reduction layers, we can evaluate larger pathological images in order to recognize the features based from cell distribution in the cancer tumor and classify the transcriptome subtypes. The network in this model is composed of 11 layers and 97,227 nodes in total. We implemented these networks based on python using TensorFlow [1] libraries, which provides various basic functions for neural networks and machine learning algorithms. We constructed and trained our network from scratch instead of applying transfer learning since the features of pathological images are not consistent with general image recognition. This time we incorporate the sparsity penalty as described in Eq. (3) to extract features and Adam algorithm for optimization.

The actual dataset is composed of pathological images of lung adenocarcinoma from the Cancer Genome Atlas (TCGA) [4, 6]. There are 409 whole slides from 230 cancer patients which are classified into three transcriptome subtypes according to their gene expression patterns. The original pathological slide images have quite high resolution of over 20,000–40,000 pixels, whose actual sizes are approximately 1–2 $\text {cm}^{2}$. We randomly clipped the original images into slices of $2048\times 2048$ image size and obtained 106,505 slices (TRU:43122, PP:27047, PI:36336) as the input data for our models.

## Results

### Visualization of filters

**Fig. 5:**
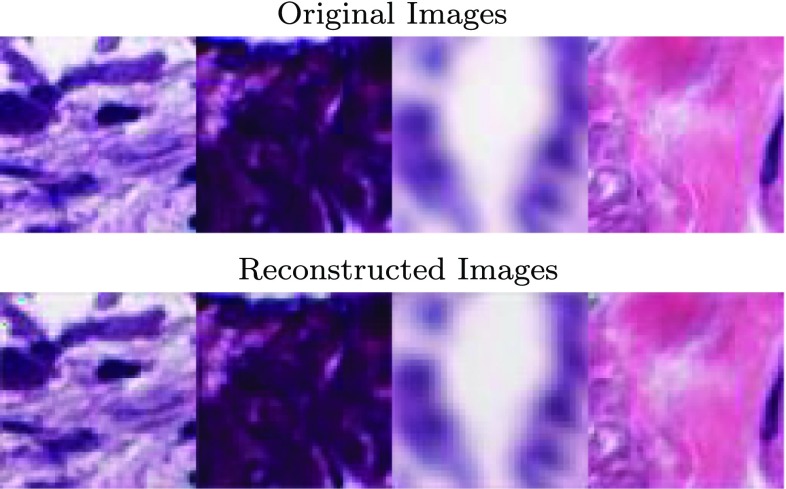
Example of the output of the autoencoder

First we look at the results of the reconstruction algorithm. While the actual slides are paired with their respective transcriptome subtypes, we use the unlabeled tiles for the autoencoder and apply the labeling for the classifier. Now, we trained three stages of autoencoder as pre-training. Figure 5 shows an example of the output of the first stage of the autoencoder. The original images here come from the general collection of images.

**Fig. 6:**
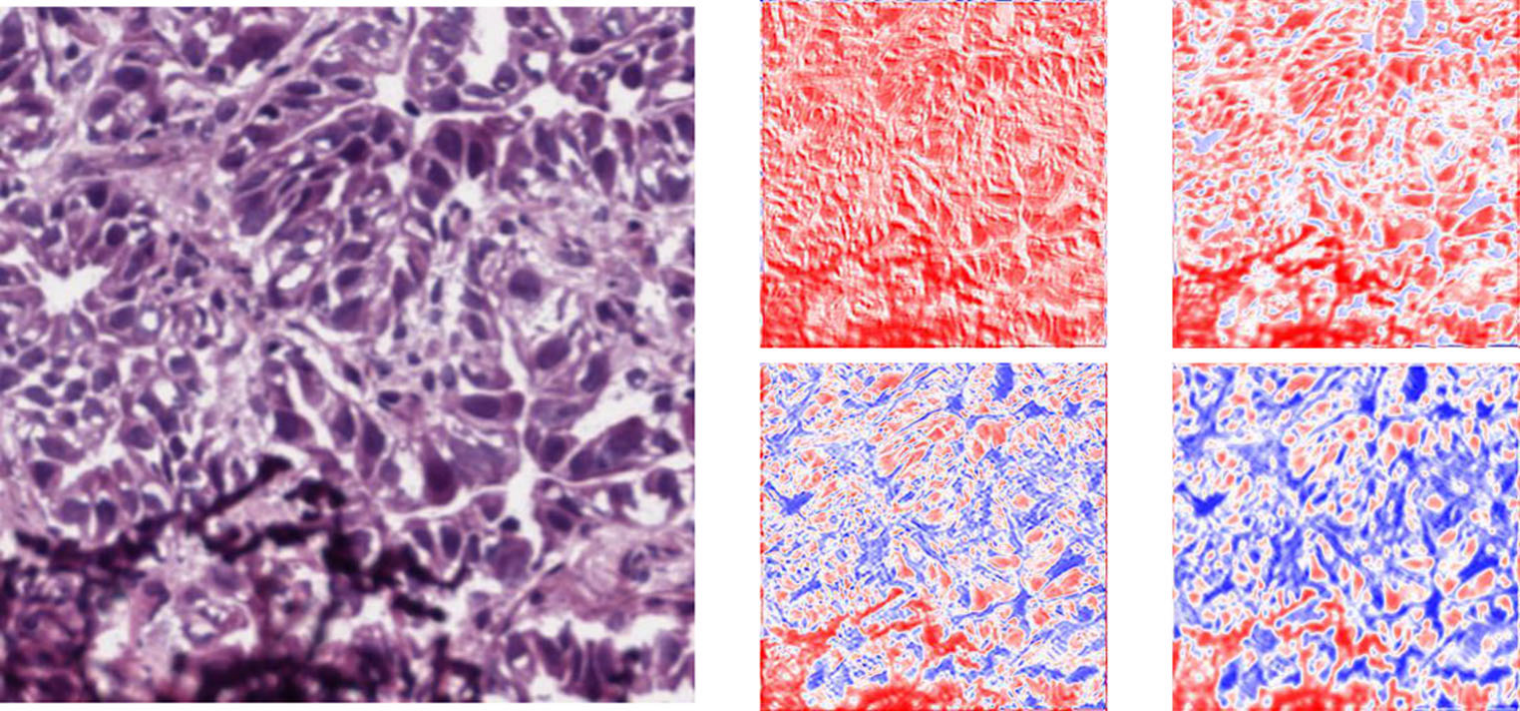
Left: input image, a sample of TRU subtype. Right: output of some encoding layers in the second autoencoder. The gradient from red to blue represents increase in signal intensity

We now look into some of the activations of the autoencoder. Though some color hue changed after reconstruction, the structural detail of the original input was recovered from compressed information of encoded layers whose resolution is one fourth of the original image, as shown in Fig. 6.

**Fig. 7:**
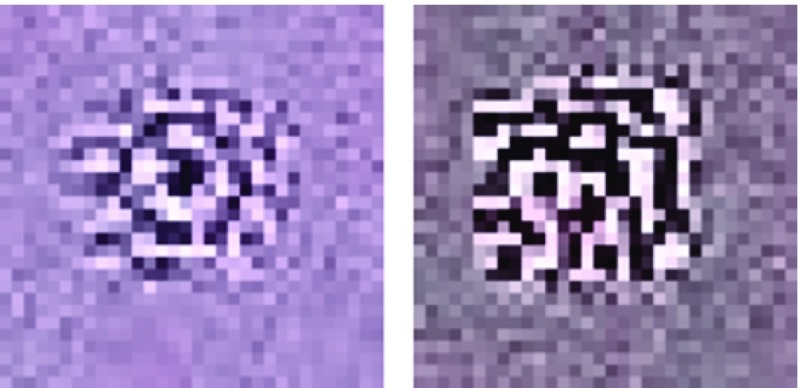
Examples of optimized local image for encoded outputs

In order to understand how the network extracts features after training, we randomly clipped the original images to generate 10,000 sample input of size $32 \times 32$ pixels. Then, we computed the output of the encoding layer and sorted them according to the value of one node in the encoding layer of the third stage. The goal of Fig. 7 is to emphasize a specific feature extracted by the autoencoder. We take the average of the pixel intensities of the top 100 encoded images based on the sorted feature activation. A sample image is then obtained representing the activation in one of the encoding layers. This represents a feature of the training image patterns. It seems that they represent different local structures of cell boundaries such as stripe- or target-like patterns.

### Internetwork comparison

We want to determine whether the convolutional filter size has an effect on the reconstruction outputs and the classification accuracy of the networks.

For the following experiments, we used $64\times 64$ images as input, and the networks follow the pipeline described in Fig. 3. First, we take a look at the reconstruction. Here we vary the convolution filter size on a standard convolutional autoencoder and a RISA network.

**Fig. 8:**
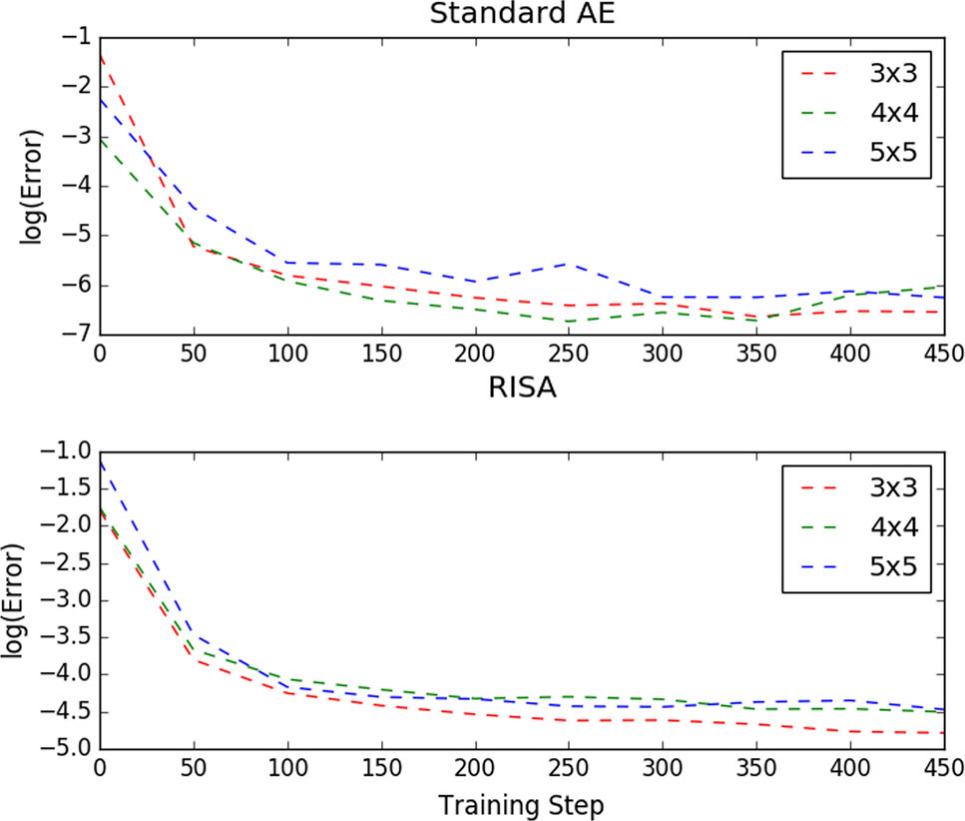
Comparison of AE and RISA training

The results are shown in Fig. 8. We take the natural logarithm of the reconstruction error over the number of training steps. Here, $3\times 3, 4\times 4$, and $5\times 5$ are the convolutional window sizes. It can be observed that performance does not vary significantly as we change the filter size. However, it can be seen, especially in the RISA network experiment, that a slight increase in reconstruction performance is brought about by a decrease in the filter size. This implies that a smaller receptive field works better for this type of task.

Next, we performed a comparison between the activated filters of each of the networks that we are working on. We take the activations of the first layer of the direct classifier, the first stage of the autoencoder, and the lone convolutional layer of the RISA network. The goal here is to determine and hopefully interpret the features extracted from each of the networks.

**Fig. 9:**
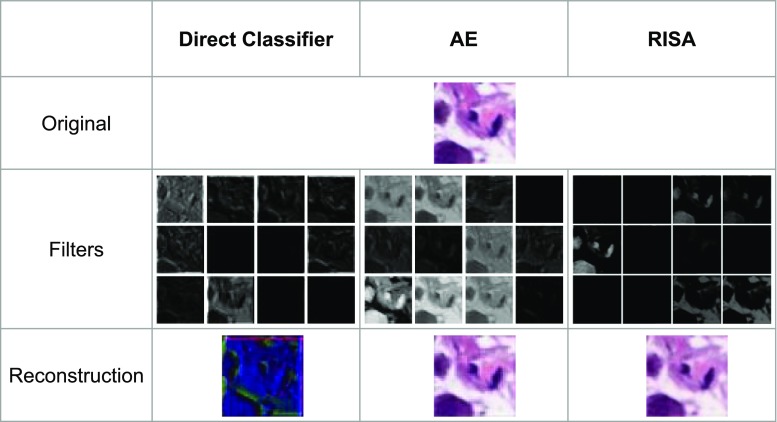
Comparison between filters and output of networks. The upper image is an original sample from PP subtype. The middle row shows outputs of some feature filters. The lower row shows the reconstructed images

In Fig. 9, we can observe several differences in the types of filters extracted. We observe that the output for the direct classifier shows some edge detection scheme through the contours in some of the filters. On the other hand, the standard autoencoder seems to emphasize shape and hue. The RISA network shows features similar to the standard autoencoder, but we also observe that some of them have paired up as part of the underlying architecture of RISA. (Note that the RISA filters were scaled to match the filters of the other networks.)

In the interest of finding the most accurate implementation of the convolutional classifier, we continue the experiment of varying the size of the input along with the convolutional filter size of the network variants. Specifically, we incorporate $32 \times 32, 64 \times 64$, and $128 \times 128$ experiments. Table 1 summarizes the accuracy of the different convolution models. In this table, we can see that, in general, there is some slight improvement in performance when we increase the filter window size.

**Table 1:** Subtype classification accuracy tables for varying networks and filter sizes $\left( \#\right)$—umber of test images

Window size	Direct class.	AE $+$ class.	RISA $+$ class.
$$32\times 32$$ (12,000)			
${3\times 3}$	73.6	76.4	52.5
${4\times 4}$	74.1	65.9	50.9
${5\times 5}$	80.5	68.8	71.3
$$64\times 64$$ (3000)			
${3\times 3}$	82.9	74.7	89.2
${4\times 4}$	87.8	79.5	62.5
${5\times 5}$	89.0	82.2	71.2
$$128\times 128$$ (750)			
${3\times 3}$	68.4	73.3	56.7
${4\times 4}$	86.4	74.3	35.9
${5\times 5}$	89.1	54.9	72.1

However, if we look at the accuracies of the RISA network, we see a different result. This can be attributed to the fact that as we increase the filter size, we have a relatively significant drop in reconstruction performance. It must be said that the effect does not seem to be drastic for the standard AE.

### Deeper networks

Using a pre-trained three-stage sparse autoencoder network, we trained to classify the transcriptome subtypes. First we confirmed the effect of block reduction. We evaluated the accuracy of the network by changing the input image size. This time, we used $128 \times {} 128, 512 \times {} 512$, and $2048 \times {} 2048$ images as input. From the results described in the previous section, we see that there is some advantage to altering the filter size of the autoencoder. As such, we use $7\times {}7, 5\times {}5$, and $3\times {}3$ for the filter size of the three stages of the autoencoder and $16\times {}16$ for the classifier.

To actually perform the classification on the $2048\times {}2048$ images, we first divide them into smaller tiles on which to apply the pre-trained convolutional autoencoder. We then concatenate the output of the final stage of the autoencoder and use it as input for the convolutional classifier.

Table 2 shows that when the input size was small, the network could not learn the difference between transcriptome subtypes very well. But as we increase the input size, more information is being read by the network, and hence, more complex features are extracted. Accordingly, the accuracy increases. It is worth noting that the number of nodes was not changed for the three experiments.

**Table 2:** Confusion matrices and accuracy for 128 px, 512 px, and 2048 px experiments

Subtype	Prediction				
	TRU	PP	PI	Total	Accuracy (%)
*Diagnosis*					
128 px					
TRU	47	32	1	80	58.8
PP	20	64	11	95	67.4
PI	16	21	44	81	54.3
Total	83	117	56	256	60.5
512 px					
TRU	54	32	0	86	62.8
PP	15	48	15	78	61.5
PI	17	16	59	92	64.1
Total	86	96	74	256	62.9
2048 px					
TRU	60	0	0	60	100.0
PP	0	49	1	50	98.0
PI	1	0	65	66	98.5
Total	61	49	66	256	98.9

## Discussion and conclusions

We aimed to implement models involving CNNs for the reconstruction and classification of lung adenocarcinoma transcriptome subtypes. The experiments using different input sizes indicate that the network requires a certain numbers of cells in the input images to recognize difference between transcriptome subtypes.

Looking at the differences of the convolutional filter output of each of the networks, we can see the features emphasized by the three variants. The convolutional network classifier outperforms the other two networks, and it can be seen that the important features have something to do with some combination of edge and hue detection. On the other hand, the autoencoder network emphasizes hue above all else. A deeper analysis of these filters is worth pursuing. Moreover, the pre-training implemented on the autoencoder-classifier networks provides several advantages like lower computational cost without a drastic effect on accuracy.

Using the pre-trained autoencoder as a feature extraction mechanism for a convolutional classifier and tiling the $2048\times {}2048$ images into individual and independent tiles paved the way for a classification algorithm involving large image input, having a 98.89% test accuracy. Even though they belong to different clusters in gene expression profiles, it was difficult to distinguish them from their morphological phenotypes since their local cell structures were not so different. In order to distinguish statistical distribution of cellular features in larger tissue images, we introduced multiple reduction layers and succeeded to classify transcriptome subtypes correctly.

This new approach will be helpful for differentiation of various tissue types, not clearly different in cell morphology, but different in cellular distribution in the tissue. This result will help the diagnosis of lung cancer for appropriate treatment, and further applications will provide us useful tools for diagnosis of various tumor types.
